# A Conceptual Fascial Memory Reset Hypothesis: Mechanobiological Insights into Stacking Fascia as an Ultrasound-Visible Structural Phenotype and the Potential Role of Fascial Hydrorelease

**DOI:** 10.3390/ijms27093720

**Published:** 2026-04-22

**Authors:** Hiroaki Kimura, Tadashi Kobayashi, Hideaki Obata

**Affiliations:** 1 Kimura Pain Clinic, Maebashi 371-0013, Japan; 2 Development of Community Healthcare, Hirosaki University Graduate School of Medicine, 5 Zaifu-cho, Hirosaki 036-8562, Japan; 3 Department of Anesthesiology, Saitama Medical Center, Saitama Medical University, Kawagoe 350-8550, Japan

**Keywords:** fascia, mechanotransduction, YAP/TAZ signaling, mechano-epigenetics, extracellular matrix remodeling, chronic pain, ultrasound-guided fascial hydrorelease, stacking fascia (proposed term)

## Abstract

This is a narrative conceptual paper, not a systematic review. Ultrasound-guided fascial hydrorelease (FHR) has been reported to provide sustained pain relief in patients with chronic musculoskeletal pain; however, its underlying biological mechanisms remain incompletely understood. In this paper, we propose the “Fascial Memory Reset Hypothesis” as an integrative framework linking mechanobiology, extracellular matrix (ECM) remodeling, peripheral nociception, microcirculatory dynamics, and ultrasound imaging findings. Mechanobiological research has demonstrated that increased tissue stiffness activates YAP/TAZ signaling, promoting fibroblast activation, ECM deposition, and mechano-epigenetic regulation. These mechanically driven processes can stabilize pathological tissue phenotypes without DNA sequence alterations. The “Fascial Memory Reset Hypothesis” proposes that targeted mechanical interventions such as FHR may partially reverse these mechanically maintained states by restoring tissue mobility and modifying stiffness-dependent mechanotransduction. We propose that “stacking fascia” (observed as layered hyperechoic bands on ultrasound) represents the macroscopic structural phenotype of mechano-epigenetic memory formed through sustained mechanical stress. Integrating molecular mechanotransduction pathways, mechano-epigenetic mechanisms, neural sensitization, and vascular factors, we propose that FHR may hypothetically partially normalize pathological fascial states by mechanically restoring tissue mobility and modifying stiffness-dependent signaling. Although direct molecular evidence of the effect of FHR in human fascia remains limited, this hypothesis provides a biologically plausible link between mechanical stress, ultrasound-visible structural alterations, and sustained clinical improvement.

## 1. Introduction

Chronic pain is a major global health burden, affecting approximately 20% of adults worldwide [1]. In recent years, structural and functional abnormalities in fascia have increasingly attracted attention as potential contributors to musculoskeletal pain conditions [2]. Deep fascia, in particular, is now recognized as an active biological tissue capable of contraction and mechanotransduction [3,4] rather than a mere passive structural element, with accumulating evidence implicating its role in chronic pain mechanisms [5]. Previous studies proposed that pathological changes in fascia—such as densification, reduced sliding capacity (hyaluronan-mediated gliding dysfunction), and altered mechanical properties—play a key role in the pathogenesis of myofascial pain syndromes [6,7]. Furthermore, impaired fascial mobility and disrupted proprioceptive function have been implicated in modulating pain perception and motor control, potentially contributing to chronic low back pain and other musculoskeletal disorders [8]. Emerging evidence links these structural alterations in deep fascia to mechanotransduction pathways, particularly the YAP/TAZ signaling axis, which regulates fibroblast activation, extracellular matrix (ECM) remodeling, and tissue stiffness in response to mechanical cues [9,10]. ECM hardening in densified fascia induces a variety of gene expression changes, particularly through the activation of YAP/TAZ and interconnected pathways, leading to profibrotic and contractile gene upregulation (e.g., COL1A1, ACTA2/α-SMA, CTGF, FN1) and mechano-epigenetic modifications that sustain pathological states [9,10,11,12,13]. These pathways may contribute to the persistence of pathological fascial states, in which repeated mechanical insults or injury induce sustained changes in fascial properties, even after resolution of the initial trigger. This sustained mechanobiological “memory” in fascia—defined as the persistent structural, biochemical, and epigenetic alterations that maintain abnormal tissue properties and nociceptive signaling long after the initial insult—may explain why many patients experience persistent pain despite the resolution of acute injury. The “Fascial Memory Reset Hypothesis” suggests that targeted interventions, such as ultrasound-guided fascial hydrorelease, may help interrupt these maladaptive mechanobiological cycles and restore normal fascial gliding and tissue homeostasis. Recent advances in ultrasound imaging have enabled in vivo visualization of fascial structures, revealing characteristic patterns of fascial thickening, increased echogenic layering, and reduced gliding. Clinically, we refer to these findings as “stacking fascia,” a term denoting hyperechoic strip-shaped lesions indicative of fascial densification and adhesion [11]. These ultrasound findings may correspond to fascial densification, as described by Stecco and colleagues [6], representing a state of increased fascial density, adhesion, and impaired gliding that contributes to persistent myofascial pain. Although the precise biological implications remain incompletely elucidated, these imaging phenotypes are increasingly regarded as indicators of compromised fascial gliding, which may sustain nociceptive signaling and perpetuate myofascial pain. In this paper, we synthesize current knowledge on fascial imaging phenotypes, mechanotransduction mechanisms, and the therapeutic potential of hydrorelease techniques in resetting pathological fascial memory. By integrating anatomical, ultrasonographic, and molecular insights, we propose a unified framework for understanding and managing chronic myofascial pain through fascia-centered therapeutic strategies.

### 1.1. Overview of Chronic Pain and FHR

Recent advances in ultrasound imaging have enabled clinicians to visualize structural variations within fascial layers. Among these, a layered and thickened fascial appearance—referred to in this paper as “stacking fascia”—is frequently observed in areas exposed to repetitive mechanical stress or chronic loading. Importantly, in this paper, stacking fascia is defined as an ultrasound-visible and clinically reproducible structural phenotype associated with altered mechanical properties of fascial tissue. Rather than representing a single molecular entity, this phenotype may reflect underlying ECM remodeling, increased tissue stiffness, and altered fascial gliding capacity.

In clinical practice, ultrasound-guided fascial hydrorelease (FHR) has emerged as a therapeutic intervention targeting these pathological fascial structures. Since its initial clinical use in the form of intrafascial saline injection in 2012, refinement under ultrasound guidance in 2014, and systematic conceptualization as FHR in 2017, the technique has evolved into a structured approach aimed at restoring fascial mobility and reducing pathological mechanical stress [8,11,14]. Unlike conventional hydrodissection or nerve blocks that primarily target neural structures, FHR focuses on releasing ultrasound-visible diseased fascial layers through mechanical separation and relaxation. FHR targets a wide range of anatomical structures, including myofascia, retinacula, joint capsules, tendon sheaths, fat pads, perineural fascia, perivascular fascia, and the ligamentum flavum [8,14].

Conventionally, the action mechanisms of FHR have been proposed to include rehydration, washout of pain substances, mechanical and chemical stimulation, nerve stimulation, improvement of extensibility, and improvement of blood flow [15]. However, the therapeutic rationale of FHR may extend beyond these simple mechanical effects. In this paper, we further examine the therapeutic mechanisms of FHR from the perspectives of mechanotransduction and mechano-epigenetics.

**Figure 1:**
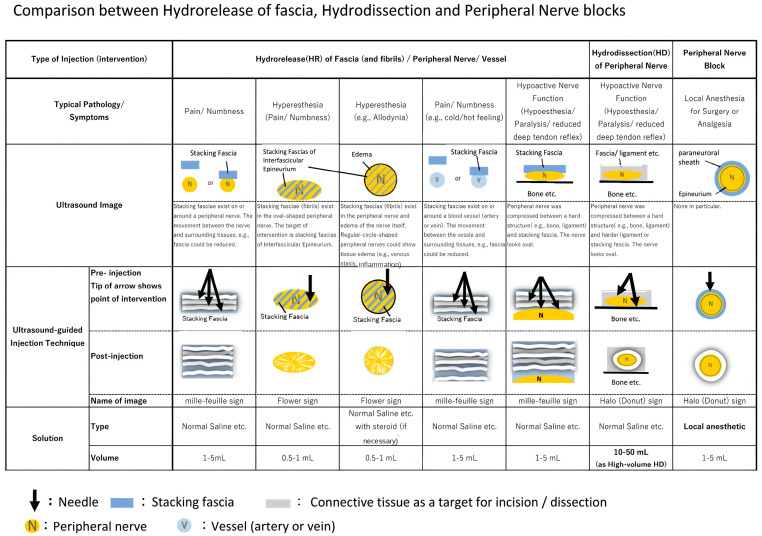
Comparison between fascial hydrorelease (FHR), hydrodissection, and peripheral nerve block, including targets, representative ultrasound images, and ultrasound-guided injection techniques. Adapted from Table 1 in [14], p. 100.

We propose that stacking fascia can be interpreted as a macroscopic structural phenotype of mechano-epigenetic memory arising from sustained mechanotransduction and YAP/TAZ signaling. From this perspective, FHR may reset pathological tissue memory by modifying both tissue mechanics and downstream biological signaling. By integrating clinical imaging findings with mechanobiology and pain physiology, this paper provides a conceptual framework linking fascial pathology, mechanotransduction, and therapeutic intervention. To illustrate the distinct characteristics of FHR in comparison with related techniques, Figure 1 presents the following three approaches:

(A) Fascial hydrorelease (FHR), performed under ultrasound guidance using primarily a 27-gauge needle to directly visualize and mechanically open diseased stacking fascia, aiming to restore fascial mobility and reduce pathological mechanical stress.
(B) Hydrodissection. Fluid is injected under ultrasound guidance to separate a nerve from surrounding connective tissue, primarily targeting peripheral nerve entrapment.
(C) Peripheral nerve block, primarily targeting the perineural space to achieve analgesia. The spread of injectate may result in partial release of the surrounding fascia; however, nerve block does not directly target stacking fascia itself.

### 1.2. Cadaver Study on the Spread of US-Guided FHR

Recent cadaveric and imaging studies have raised questions regarding the exact anatomical spread of the injectate during fascial injections, suggesting that fluid may distribute variably above or below the intended planes or even intramuscularly [16]. In contrast, FHR is performed with the aim of targeting stacking fascia under direct ultrasound visualization, seeking to mechanically open the pathological fascial layer and potentially reduce uncertainty regarding the site of therapeutic action.

Specifically, in our cadaver study, when 1 mL of dye solution was injected under ultrasound guidance into the interfascial space between the trapezius and rhomboid muscles, the solution clearly separated the two adjacent muscles and spread over a wide area. The median dye distribution area was approximately 25 cm^2^ on the deep side of the trapezius muscle and approximately 19 cm^2^ on the superficial side of the rhomboid muscle, with a statistically significant difference between the two. This difference may be attributable to the needle entry pathway and differences in the multilayered fascial structures present over the trapezius and rhomboid muscles [17]. While pigment was used for visualization in this cadaveric model, clinical injectates may exhibit comparable spread patterns under ultrasound guidance, although differences in viscosity and tissue interaction should be acknowledged. These differences in spread patterns may be influenced by needle gauge (27-gauge in our study versus 23-gauge in the study by Shiwaku et al.) and injection volume.

### 1.3. Clinical Observations and the Fascial Memory “Reset” Hypothesis

FHR is a therapeutic approach that differs from conventional hydrodissection or peripheral nerve blocks, which primarily target neural structures. Instead, FHR focuses on ultrasound-visible “stacking fascia,” characterized by a layered, hyperechoic band observed on ultrasound imaging (Figure 1). The procedure involves the injection of fluid—such as saline, Ringer’s solution, dextrose, local anesthetics, or steroids—into the pathological fascial layer with the aim of mechanically separating and relaxing the fascia, thereby potentially improving fascial extensibility and gliding. Under ultrasound guidance, the needle tip is advanced while fluid is injected to mechanically separate the thickened fascial band. Therapeutic effects are assessed through morphological changes on ultrasound imaging, improvement in range of motion, and reduction in pain intensity, allowing for structured clinical evaluation [8]. All ultrasound images presented in this study were acquired using a Konica Minolta ultrasound system with a linear probe L18-4 (4–18 MHz). Typical B-mode settings included: depth 3.5–4.0 cm, focus 2.0 cm, B-gain 27–31, dynamic range 60–65, tissue harmonic imaging on, and high-resolution mode (HRes1 or HGen2). Settings were adjusted as needed for individual patient anatomy.

In clinical practice, sustained pain relief lasting several months—beyond the expected pharmacological duration of local anesthetics or steroids—has been observed following FHR. For example, Shiwaku et al. reported that FHR performed around the posterior femoral cutaneous nerve for chronic pain after hamstring injury demonstrated an average duration of effect of 6.6 months [18]. Such prolonged effects are not fully explained by transient pharmacological action or simple mechanical adhesion release alone.

A related clinical observation concerns accessory muscles. Accessory muscles are anatomical variants not typically present in standard anatomical descriptions, including structures such as the axillary arch (Langer’s arch). Depending on their anatomical course, these muscles may compress neurovascular bundles and contribute to thoracic outlet syndrome (TOS)-like symptoms or idiopathic pain [19,20]. In our clinical experience, when present, stacking fascia has been observed to form around the axillary arch, potentially contributing to neurovascular compression and symptoms resembling frozen shoulder or TOS (see Supplementary Video S1).

**Figure 2:**
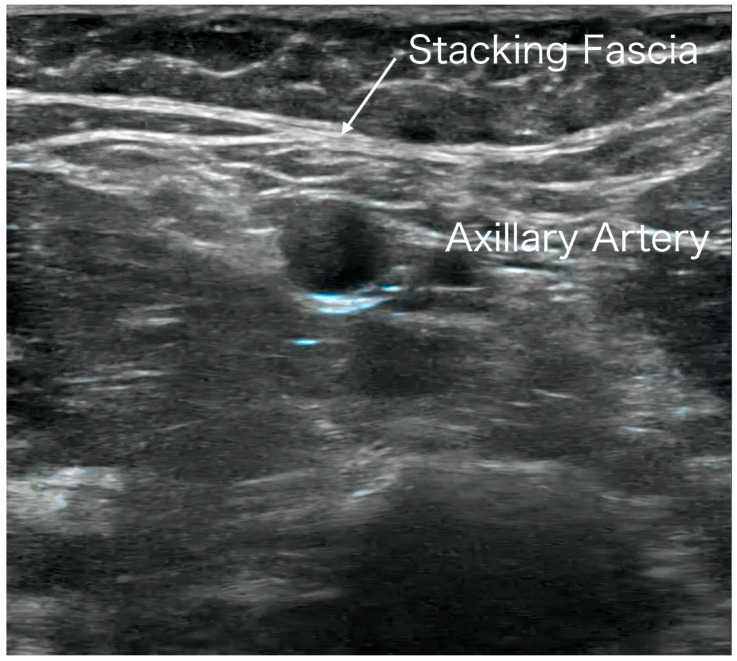
Ultrasound image of stacking fascia in the absence of an axillary arch. This figure shows stacking fascia in the axillary region of a patient without an axillary arch (Langer’s arch). A band-like hyperechoic stacking fascia can be seen superficial to the neurovascular bundle (arrow). This finding suggests that fascial memory may form at the same anatomical site through mechanical stress concentration even in the absence of accessory muscles. Release of this region is effective in conditions such as TOS and frozen shoulder (see Supplementary Video S2).

Notably, similar patterns of stacking fascia formation are frequently observed at comparable anatomical stress-concentration sites, even in the absence of accessory muscles (Figure 2; see Supplementary Video S2). This suggests that while accessory muscles themselves may contribute to symptom generation, the formation of stacking fascia in mechanically stressed regions may represent an independent and clinically relevant pathological substrate.

These observations raise questions: how might chronic mechanical stress be retained within fascial tissue, and why might FHR produce sustained therapeutic effects? To address these issues, we propose the “Fascial Memory Reset Hypothesis” as a conceptual framework.

The idea that fascia may retain a form of “memory” is not novel. Tozzi (2014) explored this possibility conceptually in “Does fascia hold memories?” [21]. However, that discussion did not identify specific molecular pathways or link the concept to defined therapeutic interventions.

Within our proposed framework, the prolonged therapeutic effects of FHR may be partially explained by alterations in the local mechanical environment following fluid injection, potentially leading to reductions in tissue stiffness and modulation of mechanotransduction pathways such as YAP/TAZ signaling. Given the established role of YAP/TAZ in the mechanosensitive regulation of fibroblast activity and fibrosis in multiple organs [22,23], changes in local mechanical stress may influence downstream epigenetic and transcriptional states.

Pirri et al. demonstrated that human thoracolumbar fascia fibroblasts exhibit YAP-dependent responses to mechanical stimulation [24], supporting the theory that mechanotransduction mechanisms are active within fascial tissue.

Based on these findings, stacking fascia—visualized as a layered, hyperechoic band on high-resolution ultrasound—may represent a macroscopic structural phenotype reflecting cumulative mechanical stress and fibroblast-driven ECM remodeling. From this perspective, FHR may influence this pathological state by modifying local tissue mechanics, thereby potentially attenuating aberrant YAP/TAZ activity and associated epigenetic alterations.

However, it must be emphasized that attenuation of YAP/TAZ activity and epigenetic changes in human fascia by FHR has not yet been verified directly. Therefore, the proposed “reset” mechanism remains hypothetical.

In this conceptual hypothesis paper, we organize findings from fascia research, mechanotransduction biology, and epigenetics into a proposed causal sequence: mechanical stress → YAP/TAZ signaling → epigenetic modification → fascial memory. Throughout this discussion, we distinguish between evidence directly demonstrated in fascia and mechanisms extrapolated from studies in other organs.

#### Key Definitions

To facilitate testability, we define the following key terms as used in this paper. Stacking fascia refers to a layered hyperechoic band-like structure observed on high-resolution ultrasound, hypothesized to represent accumulated fascial pathology. Fascial memory refers to the proposed retention of mechanical stress history within fascial tissue through YAP/TAZ-mediated epigenetic modifications. Reset refers to the hypothesized reversal of these pathological changes through targeted mechanical intervention (FHR).

### 1.4. Definition of the Fascial System

To develop the above hypothesis, it is necessary to clarify the definition of the fascial system addressed in this paper. Adstrum et al. defined fascia as “a three-dimensional continuum of fibrous connective tissue containing collagen that envelops, interconnects, and provides a whole-body network for muscles, bones, nerves, vessels, and organs” [19]. Furthermore, in an international consensus led by Adstrum et al., anatomically dissectible and distinguishable sheet-like or sheath-like connective tissues are referred to as “a fascia,” whereas the whole-body three-dimensional connective tissue network that includes these structures is termed “the fascial system,” thereby standardizing the definition and nomenclature of fascia-related structures [19]. This definition suggests that fascia can be considered not only a passive supporting tissue but also an active system that transmits mechanical stress and enables structural and functional coordination throughout the body.

### 1.5. Theoretical Basis Linking Fascia and Pain

To understand the efficacy of FHR, it is necessary to clarify the role of fascia in pain generation. Stecco A et al. demonstrated that pathological changes in fascia, including thickening, increased density, and alterations in hyaluronic acid distribution, are key contributors to pain in myofascial pain syndrome [5]. Furthermore, Ingber proposed that connective tissue functions not only as a structural support but also as a body-wide signaling network, providing a conceptual framework for understanding how localized fascial interventions may produce widespread clinical effects [25].

Previous reviews have highlighted the deep fascia as an important contributor to chronic pain, emphasizing that pathological alterations in fascial structure and function are closely associated with persistent pain conditions [2]. In addition, structural abnormalities of the thoracolumbar fascia have been reported in low back pain patients, suggesting that fascial morphology and mechanics may play a role in symptom generation [26]. These findings provide theoretical support for fascia-targeted therapeutic approaches such as FHR.

Moreover, a recent multimodal ultrasound–histological study of upper trapezius myofascial trigger points demonstrated increased deep fascial thickness, collagen lamellar hyperplasia, and elevated tissue stiffness at symptomatic sites compared with the contralateral side, all of which improved after dry needling [27]. In line with these findings, integrative perspectives on myofascial pain syndrome position fascia as a central but not isolated contributor embedded within a multifactorial framework that includes peripheral tissue pathology, central sensitization, and psychosocial influences. Accordingly, FHR may be understood as an intervention targeting peripheral fascial pathology within a broader multidimensional pain network.

## 2. Literature Review: Evidence Supporting the “Fascial Memory Reset” Hypothesis

In 2012, scientific research on fascia was still in its early stages. However, through clinical observations, we empirically recognized that pathological changes in various fascial structures—including deep fascia, retinacula, ligaments, joint capsules, tendon sheaths, fat pads, subcutaneous tissue, perineural fascia, perivascular fascia, and the ligamentum flavum—can be major sources of pain. Based on these observations, we systematized therapeutic techniques targeting these structures and established them as fascial hydrorelease (FHR) in 2017 [8,14]. As described above, the most important and distinctive feature of FHR lies in the conceptualization of stacking fascia itself as the primary source of pain. Since then, multiple independent studies have provided initial scientific support for our clinical observations. In this section, we review the evidence supporting the effectiveness of FHR and outline a conceptual framework in which stacking fascia may represent a macroscopic phenotype of mechano-epigenetic memory arising from sustained mechanotransduction and YAP/TAZ signaling. From this perspective, fascial hydrorelease (FHR) can be interpreted as a mechanical intervention that may reset pathological tissue memory.

### 2.1. Anatomical Structures Targeted by FHR and Their Scientific Validation

Although FHR targets a wide range of anatomical structures (see Section 1.1), this section focuses on perineural fascia, ligaments, and perivascular fascia, for which scientific evidence has been reported.

- Perineural Release (Nerve Hydrorelease)

The perineural fascia is one of the most important target structures of FHR. Shiwaku et al. (2025) reported that FHR applied around the posterior femoral cutaneous nerve resulted in a high pain improvement rate of 91.4%, with a mean duration of effect of 6.6 months, and proposed the concept of “perineural fascial pain” [18]. This finding supports the effectiveness of perineural release, as classified in our therapeutic framework. Langevin (2021) proposed a hypothetical model suggesting that reduced shear mobility of intermuscular fascia may contribute to proprioceptive dysfunction and myofascial pain, highlighting the potential importance of the mechanical properties of fascia in pain generation [8]. Perineural fascial release is thought to not only relieve mechanical compression of nerves but also restore the mobility of the surrounding fascia, thereby contributing to pain reduction and functional improvement. Furthermore, perineural fascial release may decompress the nervi nervorum and vasa nervorum that are compressed within the perineural environment, potentially improving nutritional blood flow and neural function.

- Ligament Release (Ligament Hydrorelease)

Ligaments are high-density connective tissues and have traditionally not been considered therapeutic targets. However, in our own study (Kimura et al., 2022), FHR applied to the coracohumeral ligament (CHL) of the shoulder joint significantly improved both range of motion and pain [11]. This represents one of the first reports demonstrating that the core concept of FHR—release of stacking fascia—is effective even in high-density connective tissues such as ligaments. In addition, Benditz et al. (2019) identified an increased presence of nociceptive nerve fibers in the ligamentum flavum of patients with lumbar spinal canal stenosis, indicating that the ligamentum flavum itself can function as a pain generator [28].

- Perivascular Release (Vessel Hydrorelease)

The perivascular fascia is also an important target of FHR. By releasing stacking fascia around blood vessels, simultaneous improvement in local blood flow and reduction in sympathetic hyperactivity may be achieved, potentially alleviating refractory symptoms such as coldness, numbness, and burning pain. Further investigation of FHR targeting perivascular fascia is warranted.

- Recent Insights into Fascia and Chronic Pain

Recent studies have further clarified the central role of fascia in chronic pain. Pirri et al. demonstrated that neuroinflammation, fibrosis, and autonomic dysregulation of fascia may contribute to pain persistence in complex regional pain syndrome (CRPS), emphasizing the importance of fascia-targeting therapies [29]. Gromakovskis et al. proposed an integrative model of myofascial pain syndrome, suggesting that the mechanical properties of fascia, inflammatory responses, and peripheral and central sensitization interact to produce chronic pain [30]. Furthermore, Stecco et al. refined the concept of the myofascial unit, demonstrating that muscle fibers, intramuscular connective tissue, deep fascia, blood vessels, and receptors function as an integrated system involved in the control of movement and pain [31]. Collectively, these recent findings support the notion that FHR is not merely an empirical therapeutic technique but rather a scientifically grounded treatment based on fascial pathophysiology.

### 2.2. Molecular and Cellular Mechanisms Supporting the Hypothesis

#### 2.2.1. Mechanotransduction, Fibrosis, and Mechano-Epigenetic Memory

- Mechanotransduction and YAP/TAZ Signaling in Fibrosis

Recent advances in mechanobiology have substantially improved our understanding of how mechanical stress alters cellular behavior. Central to this process is the YAP/TAZ signaling pathway, which functions as a key mechanosensor. The YAP/TAZ pathway has been experimentally shown to respond to mechanical stress in fibrosis across multiple organs, including the kidney, lung, liver, and heart [9,12,22,32,33,34].

ECM stiffening is primarily sensed by cells through integrin-mediated mechanotransduction pathways [35]. Increased matrix rigidity enhances focal adhesion formation and activates focal adhesion kinase (FAK) and Src signaling, followed by Rho/ROCK-dependent cytoskeletal tension [36,37]. This increase in actomyosin contractility promotes nuclear translocation of YAP/TAZ, thereby linking ECM stiffness to transcriptional regulation.

Once activated, YAP/TAZ translocate into the nucleus, induce fibroblast-to-myofibroblast transdifferentiation, and promote the production of ECM components such as collagen and fibronectin [22,32,33]. This process may create a pathological positive feedback loop in which increased tissue stiffness further amplifies YAP/TAZ activation and fibrotic remodeling.

The mechanotransduction mechanisms of YAP/TAZ, originally established by Dupont et al. [9], have since been validated in multiple organ-specific fibrosis models [32,33,34,38]. Collectively, these findings establish a feedback loop in which mechanical stress → ECM stiffening → FAK activation → YAP/TAZ nuclear translocation → mechano-epigenetic changes → further ECM production → further stiffening.

- Advancing YAP/TAZ Research in Fascial Tissue: Experimental Evidence of Mechano-Epigenetics

Mechanistic insights derived from the organ-specific studies described above (kidney, lung, and liver fibrosis models; see Section 2.1) suggest that similar pathways may operate in fascial tissue. In recent years, research directly demonstrating a relationship between mechanical stress responses and YAP/TAZ signaling in fascia has progressed rapidly. Pirri et al. (2023) were the first to demonstrate that mechanical stimulation using focused extracorporeal shock waves (fESWs) induces nuclear translocation of YAP in human thoracolumbar fascial fibroblasts, accompanied by increased expression of type I collagen (COL1A1) and hyaluronan binding protein 2 (HABP2) [24]. Importantly, this response was completely abolished by treatment with the YAP inhibitor verteporfin, indicating that YAP plays a central role in regulating mechanically induced gene expression in fascial fibroblasts. This study provided one of the first direct demonstrations of a mechanotransduction pathway in fascial fibroblasts linking mechanical stimulation to YAP activation and subsequent upregulation of ECM-related genes.

Extending these findings, Caroccia et al. (2025) demonstrated that biochemical and mechanical signals converge through YAP in human deep fascia [39]. In their study, angiotensin II (Ang II) activated YAP via the angiotensin II type 1 receptor (AT1R), inducing YAP dephosphorylation and nuclear translocation and promoting the expression of fibrosis-related genes, including COL1A1, COL3A1, and HABP2. Notably, these effects were attenuated by treatment with either the AT1R antagonist irbesartan or the YAP inhibitor verteporfin, confirming the functional importance of the Ang II–AT1R–YAP axis in fascial remodeling.

Supporting these fascia-specific findings, Jones et al. (2023) provided direct evidence that mechanical stress regulates cellular function at the epigenetic level via YAP/TAZ signaling in tendon tissue [40]. Using ATAC-seq analysis, they demonstrated that tendon unloading globally reduced chromatin accessibility, while transcription factor motif analysis identified TEAD—the transcriptional coactivator of YAP/TAZ—as the most enriched transcription factor. YAP overexpression preserved chromatin accessibility after unloading, preventing changes in 67% of chromatin regions and suppressing the induction of matrix metalloproteinase (MMP) genes. This study experimentally established a mechanistic pathway linking mechanical tension to YAP/TAZ activation, maintenance of chromatin accessibility, and regulation of gene expression, thereby defining the concept of mechano-epigenetics.

Further reinforcing this concept, Akbar et al. (2024) conducted a comprehensive multi-omics analysis of palmar fascia from patients with Dupuytren’s disease, revealing the epigenetic landscape of fascial fibrosis [41]. ATAC-seq analysis demonstrated significantly increased chromatin accessibility in diseased fibroblasts, and transcription factor motif analysis identified TEAD2—a YAP/TAZ transcriptional coactivator—as a key regulator of differentially expressed genes. Collectively, these findings demonstrate that fascial fibrosis is driven by epigenetic abnormalities involving altered chromatin accessibility and histone modifications, supporting a central role of the YAP/TAZ–TEAD axis.

- Crosstalk Between YAP/TAZ and the TGF-β/Smad Pathway

The YAP/TAZ pathway does not function in isolation but interacts closely with the transforming growth factor-β (TGF-β)/Smad pathway, a central regulator of fibrosis across multiple organs. In a renal fibrosis model, Szeto et al. (2016) demonstrated that YAP/TAZ act as mechanoregulators of TGF-β/Smad signaling [32]. Increased tissue stiffness activated YAP/TAZ, which physically interacted with Smad3 to promote its nuclear translocation, thereby amplifying TGF-β signaling and enhancing ECM production. Liu et al. (2015) similarly showed that YAP/TAZ directly drive fibroblast activation and myofibroblast differentiation in pulmonary fibrosis [22]. According to a review by Piersma et al. (2015), the TGF-β, Wnt, and YAP/TAZ pathways act cooperatively in fibrosis [12].

- Epigenetic Memory and Its Reversibility

Collectively, these studies demonstrate an integrated mechanism in which mechanical stress and biochemical signals regulate cellular function at the epigenetic level—affecting changes in chromatin accessibility and histone modifications—through the YAP/TAZ pathway, thereby driving fibrotic remodeling in fascial tissue [9,22,24,32,33,39,40,41]. A comprehensive review of YAP/TAZ signaling has emphasized their role as central hubs linking mechanical cues to persistent fibrotic remodeling. According to this study’s hypothesis, this YAP/TAZ-driven feed-forward loop serves as a molecular substrate of “fascial memory.”

Importantly, YAP/TAZ activation exhibits a context-dependent duality. Transient activation supports physiological processes such as wound healing and tissue repair, whereas chronic overactivation leads to pathological states characterized by excessive ECM production, progressive fibrosis, fascial stiffening, and reinforcement of a vicious cycle [7,9,10,32,33,42]. Accordingly, therapeutic interventions such as fascial hydrorelease (FHR) may suppress pathological “sustained activation,” potentially contributing to the normalization of YAP/TAZ activity. This may provide a plausible mechanism for interrupting the vicious cycle of fibrosis and epigenetic memory [9,22,24,32,33,39,40,41].

Notably, because the underlying DNA sequence itself remains unchanged, epigenetic states are theoretically reversible through epigenetic therapies, such as the application of DNA methylation inhibitors or histone deacetylase (HDAC) inhibitors. However, these pharmacological strategies typically require systemic administration and may be associated with serious adverse effects—including bone marrow suppression, increased infection risk, and cardiovascular complications—as well as limitations in tissue specificity [43]. This reversibility suggests that chronic pain and fibrosis may represent not irreversible structural alterations but resettable functional states, providing a theoretical rationale for therapeutic intervention. Furthermore, Alvarado et al. (2015) proposed that pain memory may be maintained not only in peripheral tissues but also through DNA methylation changes in the prefrontal cortex (PFC), a brain region involved in the cognitive and emotional processing of pain, potentially contributing to pain chronification and comorbid conditions such as depression and anxiety [44].

In contrast, physical interventions such as FHR may act locally at the lesion site and modulate the epigenetic state of target tissues by reducing local tissue stiffness. Specifically, this could involve YAP/TAZ inactivation through decreased mechanical tension and subsequent normalization of ECM production, as well as normalization of epigenetic changes in the PFC through reduced peripheral nociceptive input. However, these mechanisms remain speculative at present and require direct experimental verification in future studies.

#### 2.2.2. ECM Stiffness, Peripheral Nociception, and Mechanical Pain Mechanisms

Beyond their role in mechano-epigenetic regulation described above, increased ECM production and tissue stiffening may also directly influence peripheral nociception through biomechanical mechanisms.

- Tissue Stiffness and Neural Function

Lantoine et al. (2016) quantitatively examined how substrate stiffness affects neuronal function [45]. Primary cortical neurons were cultured on two substrates with identical biochemical properties except for a 100-fold difference in Young’s modulus (stiffness), and their growth dynamics and electrophysiological activity were compared. Neurons on the stiffer substrate showed a two-fold increase in presynaptic density and significant increases in action potentials and miniature synaptic currents. This study demonstrated that matrix stiffness is a key parameter regulating neuronal network development, synapse density, and electrophysiological activity, revealing that the mechanical properties of the matrix directly control neuronal function.

- Role of the Mechanosensitive Ion Channel Piezo2

Murthy et al. (2018) showed that the mechanosensitive ion channel Piezo2 mediates sensitized mechanical pain induced by inflammation and nerve injury [46]. Optogenetic activation of Piezo2-expressing sensory neurons induced nocifensive behavior, whereas mice lacking Piezo2 in caudal sensory neurons displayed impaired protective responses to mechanical stimuli. Notably, in models of capsaicin-induced inflammation and partial sciatic nerve injury, Piezo2-deficient mice completely lacked both punctate and dynamic mechanical allodynia. These findings indicate that Piezo2 is an essential molecular mediator of mechanical allodynia, a state in which normally innocuous tactile stimuli are perceived as painful.

Obeidat et al. (2023) reported that nociceptor-specific Piezo2 knockout mice are protected from mechanical sensitization in an osteoarthritis model, suggesting that Piezo2 plays a critical role in post-inflammatory mechanical hypersensitivity [47]. In a complementary investigation, Freeberg et al. (2025) showed in a lung fibrosis model that stiff substrates activate Piezo2 in fibroblasts and promote their differentiation into myofibroblasts [48].

- Rich Innervation of Fascia and Diverse Nociceptive Mechanisms

Fascial tissue is richly innervated not only by Piezo2-expressing fibers but also by a variety of nociceptors. Mense [42] conducted a detailed analysis of thoracolumbar fascia innervation, as previously characterized quantitatively by Tesarz et al. [49], and reported that the fascia contains abundant free nerve endings expressing nociceptive markers such as TRPV1 (capsaicin receptor), substance P (SP), and calcitonin gene-related peptide (CGRP) [42]. Moreover, a systematic review by Suarez-Rodriguez et al. (2022) showed that fascia is predominantly innervated by nociceptive neural networks and that the number of nociceptors increases following inflammation or injury [50].

- Role of ECM Stiffness in Persistent Postoperative Pain

Synthesizing these experimental findings, Plaut (2025) proposed a theoretical framework for the role of ECM stiffness in persistent postoperative pain [51]. In this hypothesis, ECM remodeling after surgery increases tissue stiffness, and the stiffened ECM generates mechanical tension and compression on sensory nerves and neuromas, thereby producing persistent nociceptive signaling. Importantly, Plaut suggested that modifying the stiffened ECM could represent a therapeutic approach for refractory pain.

- Integrated View: From Mechanical Stress to Pain

Taken together, these studies outline the following pathway:

Mechanical stress → YAP/TAZ-mediated ECM remodeling (as described in Section 2.2) → tissue stiffening → mechanical tension and compression on sensory nerves → activation of nociceptive pathways (see Section 3.2) → nociceptor sensitization → development of mechanical allodynia.

Therefore, softening of the ECM by FHR is theoretically consistent with Plaut’s model and may reduce not only mechanical signals mediated by Piezo2 but also nociceptive signals mediated by chemically sensitive receptors such as TRPV1 and inflammatory mediators, thereby exerting multifaceted therapeutic effects.

Although YAP/TAZ-mediated mechanotransduction is a central molecular mechanism underlying fibrosis and tissue stiffening, the biological consequences of ECM rigidity likely extend beyond intracellular signaling pathways. Increased tissue stiffness may directly influence peripheral nociception through mechanical compression, altered mechanosensitive ion channel activity, and enhanced neural sensitization. Therefore, FHR may exert therapeutic effects at multiple biological levels, potentially acting through both mechano-epigenetic modulation and direct mechanical normalization of the peripheral nociceptive environment.

Taken together, these findings suggest that ECM stiffening should not be interpreted solely as a downstream consequence of intracellular mechanotransduction but rather as a central integrative node linking multiple biological processes. Increased tissue stiffness may simultaneously promote mechano-epigenetic signaling (e.g., YAP/TAZ activation), direct biomechanical activation of peripheral nociceptors, and alterations in local microcirculation. From this perspective, stacking fascia may represent a macroscopic manifestation of these converging processes rather than a single molecular entity. Accordingly, fascial hydrorelease (FHR) may exert therapeutic effects through multi-level mechanisms by mechanically modifying the tissue environment, thereby influencing cellular signaling, neural sensitization, and vascular dynamics in an integrated manner.

#### 2.2.3. Microcirculatory Dysfunction, Ischemic Pain, and Neurovascular Mechanisms

Beyond the mechano-epigenetic regulation and peripheral nociceptive mechanisms described above, alterations in microcirculation and neurovascular interactions may represent an additional and clinically important pathway contributing to chronic pain associated with fascial pathology. Fascial stiffening and ECM remodeling may impair local tissue perfusion by increasing mechanical tension around blood vessels, reducing the microvascular mobility, and altering the neurovascular coupling within the fascial environment. Such microcirculatory disturbances can promote local ischemia, metabolic stress, and the accumulation of algesic substances, thereby contributing to ischemic muscle pain and persistent nociceptive signaling.

Ischemia-induced muscle pain has been extensively studied, demonstrating that reduced perfusion and accumulation of metabolic byproducts can directly activate deep tissue nociceptors and sustain persistent pain signaling [52,53]. Groups III and IV muscle afferents are particularly sensitive to ischemia-related metabolites, including protons, ATP, and inflammatory mediators, providing a physiological basis for ischemic pain and mechanical hypersensitivity [53]. These mechanisms suggest that impaired local circulation associated with fascial stiffening may contribute not only to biochemical sensitization but also to sustained nociceptive input from deep tissues.

Importantly, arteries and veins frequently run together within shared fascial compartments, suggesting that pathological stiffening of perivascular fascia may simultaneously affect vascular flow dynamics and adjacent neural structures. From this perspective, mechanical interventions targeting fascial stiffness may influence not only intracellular signaling pathways such as YAP/TAZ-mediated mechanotransduction and peripheral mechanosensitive nociception but also local hemodynamics and neurovascular function. Clinical observations and emerging theoretical frameworks suggest that restoration of fascial mobility may improve blood flow, reduce local tissue pressure, and normalize neurovascular interactions.

Accordingly, fascial hydrorelease (FHR) may exert therapeutic effects through a multi-level mechanism that includes modulation of microcirculation and relief of ischemia-related pain, thereby complementing the mechano-epigenetic and nociceptive pathways discussed in Section 2.2.1 and Section 2.2.2. This integrative perspective supports a model in which fascial interventions influence pain through combined effects on cellular signaling, mechanical nociception, and vascular–metabolic regulation.

In Section 2.2.1, Section 2.2.2 and Section 2.2.3, mechano-epigenetic regulation, peripheral nociceptive mechanisms, and microcirculatory dysfunction have been presented as interconnected yet distinct pathways. Importantly, it is unlikely that these processes operate independently in vivo. Instead, chronic mechanical stress may simultaneously influence intracellular signaling pathways, ECM remodeling, neural sensitization, and local hemodynamics, resulting in a multi-scale pathological state within fascial tissue.

Building on the mechanobiological and neurovascular interactions described above, the following section integrates these multi-scale processes into a unified conceptual model explaining both the development of stacking fascia and the potential therapeutic mechanisms of fascial hydrorelease (FHR).

**Figure 3:**
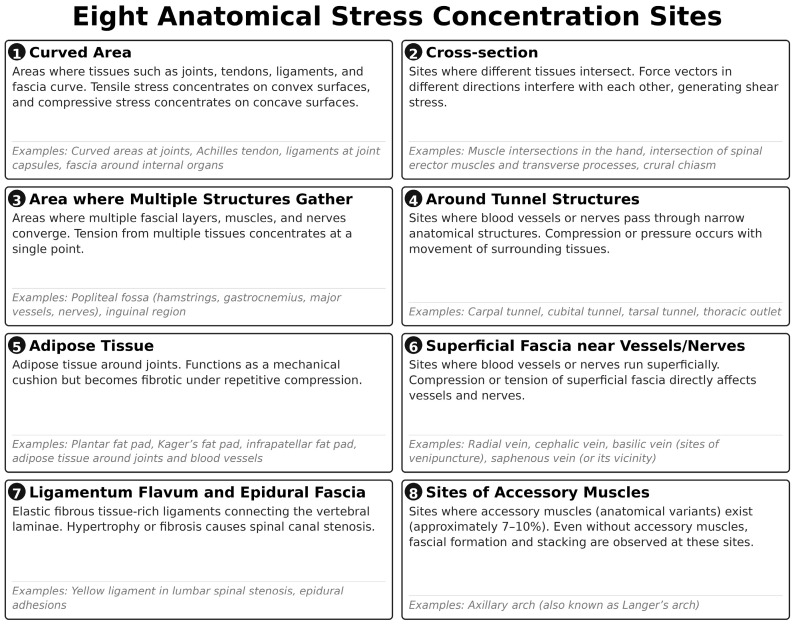
Sites with a predilection for fascial memory formation. Schematic illustration of eight anatomical categories of sites in which stacking fascia preferentially develops due to concentrated mechanical stress.

Fascial memory is not formed uniformly throughout the body. Clinical observations suggest that it develops preferentially at specific anatomical sites in which mechanical stress tends to concentrate. We categorized these sites into eight groups (Figure 3):

Curved regions of tissues: Sites where muscles or nerves curve (e.g., the curvature of the iliopsoas muscle).

Crossing points of tissues: Sites where different tissues cross (e.g., crossing of extensor tendons at the wrist).

Convergence zones of multiple tissues: Sites where multiple muscles or tendons converge (e.g., the anterior aspect of the hip joint).

Peritubular regions: Sites where nerves or vessels pass through tunnels (e.g., carpal tunnel, tarsal tunnel).

Fat pads: Periarticular adipose tissues (e.g., the infrapatellar fat pad).

Neurovascular superficial courses: Sites where vessels and nerves run just beneath the superficial fascia (e.g., the axillary region).

Ligamentum flavum and epidural space: Interspinous ligaments and epidural fascia.

Predilection sites for accessory muscles: Regions where variant muscles frequently occur, such as the axillary arch.

## 3. Integrated Model of Stacking Fascia and Therapeutic Mechanisms of FHR

Ultrasound-guided hydrodissection was originally developed as a technique to separate adhesions around nerves by injecting fluid. Cass described hydrodissection as reducing pain by mechanically separating perineural scar tissue and restoring nerve mobility [54]. In a retrospective study, Lam et al. reported that deep nerve hydrodissection with 5% dextrose water was effective for chronic pain in the upper body and trunk [55].

These studies indicate that hydrodissection is not merely the injection of local anesthetics but a treatment that alters tissue structure through the mechanical action of fluid. Our FHR extends this concept beyond perineural tissue to all types of stacking fascia, including those around ligaments, vessels, and muscles.

As established in Section 2, mechanical stress activates the YAP/TAZ pathway in multiple organ systems and, importantly, in human fascial tissue itself (Section 2.2), where it induces epigenetic changes that sustain fibroblast-to-myofibroblast transition and excessive ECM production. In other words, fascia has the capacity to “remember” chronic mechanical stress as epigenetic remodeling.

Building on this established evidence, in this section, we integrate clinical observations into a theoretical framework. Specifically, we explain where and how fascial memory is formed (Section 3.1 and Section 3.2) and how it can be targeted therapeutically (Section 3.3).

### 3.1. Physical Substrate of Fascial Memory: Anatomical Stress-Concentration Sites and Stacking Fascia

As described in Section 1.3, stacking fascia is proposed as the macroscopic structural correlate of the mechanobiological processes outlined in Section 2. At anatomical stress-concentration sites, these processes converge to produce the ultrasound-visible layered structures.

At these anatomically vulnerable regions, persistent mechanical stress may induce YAP/TAZ-mediated epigenetic changes, leading to fibroblast phenotype alteration, excessive ECM deposition, and tissue stiffening. These processes become visible on ultrasound as localized hyperechoic band-like structures referred to as stacking fascia (Figure 2).

Rather than a simple anatomical variation, stacking fascia may be interpreted as a physical manifestation of fascial memory—a structural imprint of chronic mechanical stress.

Importantly, these sites overlap with regions prone to postoperative pain chronification. The chronification of postoperative pain involves peripheral and central sensitization, as well as the formation of persistent pain memory [24,44,53,56]. By mechanically releasing stacking fascia, FHR may reduce pathological mechanotransduction, suppress YAP/TAZ activation, and potentially reset epigenetic tissue memory.

### 3.2. Mechanobiological Pathway Leading to Fascial Memory Formation

Fascial memory is unlikely to arise from a single isolated mechanism; rather, it appears to emerge from the cumulative interaction of mechanical, cellular, and neurovascular processes over time. Building upon the mechanistic components described in Section 2, we propose a multi-scale pathway in which sustained mechanical stress induces progressive structural and functional alterations within fascial tissue.

First, repetitive mechanical loading at anatomically vulnerable sites (see Section 3.1) activates mechanotransduction pathways, including the YAP/TAZ signaling cascade detailed in Section 2.2.1, potentially promoting excessive ECM deposition and increased tissue stiffness [8,11,16,21,22,23,24,47,48].

Second, structural remodeling of the fascial layers leads to densification, adhesion formation, and reduced sliding capacity [6,7,13], progressively creating a positive feedback loop of increasing stiffness and mechanical stress concentration.

Third, neurovascular interactions as described in Section 2.2.2 and Section 2.2.3 likely contribute to the establishment of fascial memory: mechanical compression may sensitize peripheral nociceptors and impair microcirculation, reinforcing both structural and functional changes [16,42,44,50,51,52,53,56].

In summary, stacking fascia can be understood as an emergent phenotype reflecting the convergence of the mechanobiological, nociceptive, and microcirculatory mechanisms detailed in Section 2, Section 3.1 and Section 3.2, rather than a single pathological entity.

### 3.3. Integrated Therapeutic Mechanisms of Fascial Hydrorelease (FHR)

**Figure 4:**
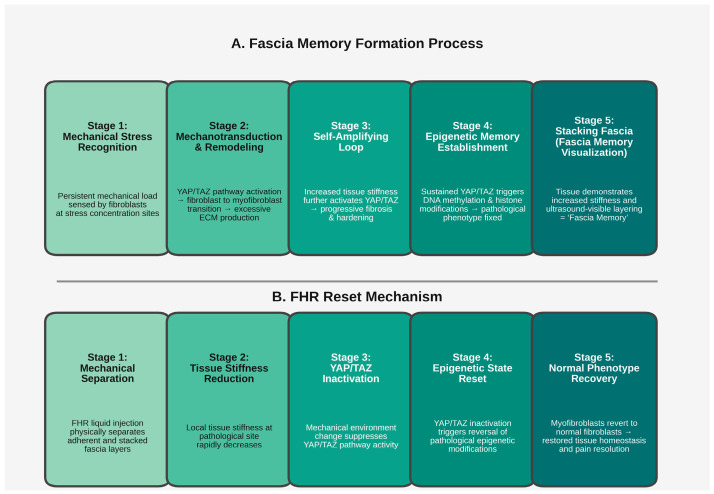
Fascial memory formation and FHR reset mechanism. (**A**) Five-stage formation process showing how persistent mechanical stress is converted into epigenetic memory through YAP/TAZ signaling, resulting in stacking fascia. (**B**) Proposed five-stage FHR mechanism that reverses the pathological process by reducing tissue stiffness and suppressing YAP/TAZ-mediated epigenetic memory, leading to recovery of a normal tissue phenotype.

Based on the multi-scale model proposed above, fascial hydrorelease (FHR) may exert therapeutic effects through several interconnected mechanisms rather than a single pathway. Although illustrated as sequential stages in Figure 4 for conceptual clarity, these mechanisms likely occur in parallel and interact dynamically in vivo. We propose that FHR acts simultaneously at mechanical, cellular, neurophysiological, and microcirculatory levels, potentially contributing to the reversal of fascial memory.

First, from a biomechanical perspective, FHR mechanically separates densified fascial layers and restores sliding capacity between tissue planes. Existing fascial studies have demonstrated that pathological densification and reduced shear strain are associated with chronic pain conditions, suggesting that restoration of fascial mobility may normalize tissue biomechanics [6,7,13]. The injection of fluid under ultrasound guidance may also reduce localized mechanical stress concentration, interrupting the positive feedback loop of stiffness-induced mechanotransduction.

Second, FHR may influence mechano-epigenetic regulation. By reducing tissue stiffness and mechanical tension, FHR may suppress pathological YAP/TAZ activation (see Section 2.2), thereby hypothetically contributing to normalization of the fibroblast phenotype and epigenetic regulation [11,21,22,23,24,47,48].

Third, FHR may modulate peripheral nociception through mechanical and biochemical pathways. Mechanical decompression of fascial compartments may reduce peripheral nociceptor activation (see Section 3.2) [42,50,51,52,53]. Additionally, washout of inflammatory mediators and algesic substances may further reduce nociceptive signaling.

Fourth, restoration of local microcirculation is an additional potential mechanism. Mechanical release of perivascular fascia may improve vascular mobility and perfusion, reducing ischemia-related pain and metabolic stress. Previous studies on muscle afferents and ischemic muscle pain suggest that reduced perfusion and accumulation of metabolic byproducts can activate Groups III and IV afferent fibers, contributing to persistent pain [44,53,56]. Clinical observations indicate that fascial release may improve pulsatile blood flow and reduce tissue pressure, supporting a neurovascular mechanism of action.

Considering these factors together, FHR may function as an integrative intervention acting across multiple biological scales. Rather than targeting a single molecular pathway, FHR may simultaneously modulate tissue biomechanics, mechanotransduction signaling, peripheral nociceptive mechanisms, and microcirculatory dynamics. From this perspective, FHR may contribute to resetting pathological fascial memory by mechanically normalizing the local tissue environment.

This conceptual model is summarized schematically in Figure 4, illustrating both the multi-stage formation of fascial memory and the proposed sequential reset mechanism induced by FHR.

This reset mechanism is, at present, based on theoretical inference and requires verification by future experimental studies. In particular, analysis of YAP/TAZ activity and epigenetic markers in fascial tissue before and after FHR will be crucial for testing this hypothesis. Nonetheless, the proposed mechanism provides a key explanation for the long-term therapeutic effects of FHR that extend beyond the pharmacological duration of local anesthetics and steroids, and it is consistent with the clinical evidence presented in the next section.

These stages are conceptual and may occur in parallel rather than strictly sequentially.

## 4. Clinical Evidence

The fascial memory hypothesis is supported not only by molecular biological theory but also by observations made in daily clinical practice. Clinical findings across various musculoskeletal conditions suggest that pathological alterations in fascial structure and mechanical properties contribute to chronic pain states and that fascia-targeting interventions produce meaningful therapeutic effects [2,5,6,7,13,17].

### 4.1. CRPS: An Extreme Clinical Manifestation of Fascial Memory

CRPS is a complex, refractory condition that often develops after minor trauma and is characterized by disproportionate severe chronic pain, allodynia, edema, autonomic dysregulation, and motor dysfunction [57]. Current CRPS research has identified multiple pathophysiological mechanisms, including aberrant inflammatory responses with peripheral cytokine release, autoantibody production, central sensitization, and maladaptive neuroplasticity [57,58,59]. CRPS is therefore understood as a multifactorial condition that cannot be attributed to a single mechanism.

In clinical practice, we have encountered patients who developed CRPS after procedures such as venipuncture, in whom ultrasound demonstrated localized fascial changes around the puncture site (e.g., the cephalic vein). Fascial hydrorelease (FHR) targeting these regions was associated with pain relief and functional improvement in some cases (Supplementary Video S3). However, it should be noted that CRPS often involves significant limb swelling and extensive tissue changes, and our observations are limited to a subset of cases.

These clinical observations do not imply that fascial pathology is the primary or sole mechanism underlying CRPS. Rather, fascial adhesion and remodeling may represent one contributing factor within the broader multifactorial pathophysiology of CRPS, which includes peripheral and central sensitization, neuroinflammation, autoimmune mechanisms, and autonomic dysfunction [57,58,59].

From the perspective of the fascial memory hypothesis, CRPS may involve a component of sustained mechanotransduction and tissue remodeling that could contribute to the perpetuation of symptoms. However, this interpretation should be understood as one possible contributing mechanism among many, and further research is needed to clarify the role of fascial pathology in CRPS pathogenesis.

### 4.2. Clinical Observations in Common Chronic Pain

Beyond extreme conditions such as CRPS, fascial pathology may also play an important role in more common chronic musculoskeletal pain conditions. Clinical studies cited in this paper suggest that FHR targeting various forms of stacking fascia—including perineural fascia, ligaments, and perivascular fascia—can produce long-lasting improvements in pain and function [11,16].

For example, sustained clinical improvements have been reported following FHR targeting perineural fascia [18] and the coracohumeral ligament [11] (see Section 1.1 and Section 2.1 for details).

These findings support the applicability of the fascial memory hypothesis presented in Section 3, not only to extreme pathological states but also to common chronic and postoperative pain frequently encountered in routine clinical practice. Mechanical normalization of fascial tissue may simultaneously influence tissue biomechanics, mechanotransduction signaling, and neurovascular function, thereby contributing to sustained therapeutic effects.

Considering the above factors together, stacking fascia may represent a clinically relevant structural phenotype associated with chronic pain, and targeted mechanical release may help restore physiological tissue function.

To clarify the level of evidence supporting each key claim in this paper, the following table classifies the cited studies as “Direct,” “Indirect,” or “Hypothetical” (Table 1).

## 5. Discussion

In this paper, stacking fascia refers to an ultrasound-visible layered structural phenotype characterized by reduced fascial sliding and localized densification.

Within this framework, pathological fascial states are interpreted not as static structural abnormalities but as dynamic, mechanically maintained conditions that remain biologically modifiable. The concept of fascial memory describes persistent structural and functional adaptations induced by chronic mechanical stress, whereas the reset hypothesis refers to partial normalization of mechanically maintained pathological states rather than complete reversal. Fascial hydrorelease (FHR) may function as a mechanical intervention that helps interrupt maladaptive mechanobiological feedback loops by restoring tissue mobility, reducing pathological stiffness, and modulating local neurovascular environments.

The following sections integrate mechanobiological evidence, chronic pain mechanisms, and therapeutic implications to discuss how stacking fascia may be understood within a multi-scale framework and how mechanical interventions may influence both tissue structure and biological signaling networks.

### 5.1. Multi-Pathway Model of Chronic Pain in Fascial Pathology

Chronic pain associated with fascial pathology is unlikely to be explained by a single mechanism. Instead, a multi-scale interaction between several biological processes is mechanistically plausible:

- Direct biomechanical activation of peripheral nociceptors;
- Neurovascular interactions and microcirculatory dysfunction;
- Accumulation of metabolic and inflammatory mediators;
- Mechano-epigenetic regulation mediated by mechanotransduction pathways such as YAP/TAZ signaling.

Fascial tissues are richly innervated by nociceptive fibers (see Section 3.2), suggesting that increased ECM stiffness may directly influence pain perception through mechanical stimulation of sensory pathways [42,50,51,52,53]. Additionally, ischemia-related mechanisms involving Groups III and IV muscle afferents may contribute to persistent pain under conditions of reduced local perfusion or increased tissue pressure [44,53,56].

From this perspective, stacking fascia may represent an emergent structural state arising from the convergence of multiple biological processes rather than a discrete disease entity.

### 5.2. Therapeutic Implications of Fascial Hydrorelease

Fascial hydrorelease (FHR) may be a mechanical intervention capable of influencing multiple levels of this multi-scale system simultaneously. By mechanically separating densified fascial layers and restoring tissue mobility, FHR may help reduce localized mechanical stress concentration and contribute to the interruption of pathological feedback loops.

The potential mechanisms, as proposed pathways, of FHR’s effects may include

1. Restoration of fascial sliding and biomechanical normalization [6,7,13];
2. Reduction in sustained mechanotransduction signaling by decreasing tissue stiffness [21,22,23,24];
3. Decreased activation of mechanosensitive nociceptors [42,50,51,52,53];
4. Improvement of local microcirculation and tissue perfusion, potentially reducing ischemia-related pain [44,53,56].

The long-lasting clinical effects observed following FHR suggest that its therapeutic action may extend beyond transient pharmacological effects, potentially reflecting mechanical modulation of mechanically maintained pathological tissue states.

### 5.3. Conceptual Implications of the Fascial Memory Reset Hypothesis

The Fascial Memory Reset Hypothesis extends the concept of fascial memory by proposing that mechanically induced pathological tissue states are dynamic and potentially reversible under appropriate mechanical conditions. The term “memory” refers not to the literal storage of information but to persistent structural and functional adaptations mediated by sustained mechanotransduction, ECM remodeling, and mechano-epigenetic regulation.

Within this framework, fascial tissues are viewed as adaptive biological systems capable of undergoing both adaptive and maladaptive remodeling in response to mechanical impacts. The reset hypothesis emphasizes that normalization of pathological mechanical environments may partially restore physiological tissue regulation.

From this perspective, FHR may be conceptualized not merely as a mechanical separation technique but as a targeted intervention that could help modulate mechanobiological states by reducing tissue stiffness, restoring fascial sliding, and altering local mechanical forces. These changes may hypothetically disrupt feed-forward cycles of mechanotransduction and potentially influence fibroblast behavior and tissue homeostasis.

This framework integrates molecular mechanotransduction, mechano-epigenetic regulation, tissue biomechanics, peripheral nociception, and clinical imaging findings into a unified model and provides a mechanistically plausible explanation for the long-lasting clinical effects of mechanical therapies.

### 5.4. Limitations and Future Directions

Several limitations of this paper should be acknowledged. First, the proposed mechanisms are based on the integration of experimental findings and clinical observations rather than direct causal demonstration in fascial tissue. Second, the relationship between stacking fascia and specific molecular pathways remains inferential and requires further validation.

Third, there is no histological confirmation that ultrasound-visible stacking fascia corresponds to densification and ECM changes at the tissue level. Fourth, there is no direct evidence linking US-visible stacking fascia to mechanotransduction pathways such as YAP/TAZ activation in human fascia specifically. Fifth, the absence of pre- and post-FHR molecular data in human fascial tissue means that the proposed reset mechanism remains largely theoretical. Sixth, standardized quantitative ultrasound criteria for defining and measuring stacking fascia remain lacking, limiting reproducibility and comparability across studies. Finally, the mechanistic evidence is based on small observational series rather than large controlled trials, necessitating caution in interpreting causality and generalizing findings to broader patient populations.

Future studies should investigate changes in YAP/TAZ activity, ECM composition, and epigenetic markers before and after FHR to test the proposed reset mechanism. Specifically, the following parameters are proposed as validation targets: (1) YAP nuclear localization ratio in fascial fibroblasts; (2) COL1A1 and ACTA2 gene expression levels; (3) shear wave elastography stiffness values; (4) dynamic ultrasound assessment of fascial layer sliding; and (5) epigenetic markers including H3K27me3 and DNA methylation patterns. It should be noted that none of these parameters have been directly measured in the context of FHR to date; the absence of such evidence constitutes a key limitation of the current hypothesis. Additionally, standardized ultrasound criteria for identifying stacking fascia and quantitative biomechanical measurements will be essential to improving reproducibility and clinical translation. Alternative explanations, such as transient fluid redistribution or non-specific mechanical effects, cannot be excluded and should be considered in future investigations.

The conventional action mechanisms described in Section 1.1 (rehydration, washout of pain substances, mechanical and chemical stimulation, nerve stimulation, improvement of extensibility, and improvement of blood flow [15]) may themselves serve as alternative explanations for the observed effects of FHR. In addition, placebo effects, as well as natural course and spontaneous resolution of symptoms, should also be considered. Distinguishing the contribution of the proposed fascial memory reset mechanism from these alternatives will require carefully designed controlled studies.

## 6. Conclusions

As proposed in this paper, stacking fascia is hypothesized to represent a macroscopic structural phenotype reflecting cumulative mechanobiological adaptation to chronic mechanical stress. By integrating mechanotransduction signaling, nociceptive mechanisms, and microcirculatory factors, the Fascial Memory Reset Hypothesis provides a novel conceptual framework for understanding chronic pain and therapeutic intervention.

Within this framework, fascial hydrorelease (FHR) may function as a mechanical intervention that contributes to normalization of local biomechanics, modulation of mechanotransduction pathways, and improvement of neurovascular function. Although the proposed mechanisms require further experimental validation, this hypothesized integrative model offers, as a proposed conceptual framework, a biologically plausible link between molecular processes, ultrasound-visible structural changes, and clinical outcomes.

Future research focusing on mechanobiological markers, epigenetic regulation, and quantitative imaging will be essential to verify the reset mechanism and to establish evidence-based therapeutic strategies targeting pathological fascial remodeling.

## Figures and Tables

**Table 1:** Evidence classification for key claims presented in this paper.

Claim/Concept	Evidence Category	Key Support
Fascia is richly innervated with nociceptors and mechanoreceptors	Direct	Mense [42]; Tesarz et al. [49]; Suarez-Rodriguez et al. [50]
Fascial gliding impairment observable on dynamic ultrasound	Direct (observational)	Shiwaku et al. [16,18]
FHR produces immediate symptomatic relief	Direct (observational)	Kimura et al. [11]; Shiwaku et al. [16,18]; JNOS [15]
Stacking fascia visible as hyperechoic bands on ultrasound	Direct (observational)	Clinical observations (this paper)
YAP/TAZ mechanotransduction in fascial fibroblasts	Direct (fascial tissue)	Pirri et al. [24]; Caroccia et al. [39]
YAP/TAZ mechanotransduction regulates fibroblast phenotype	Indirect (non-fascial cell studies)	Dupont et al. [9]; Piccolo et al. [10]; Liu et al. [22]
Epigenetic changes encode persistent mechanical memory	Indirect (other tissues)	Jones et al. [40]; Akbar et al. [41]; Alvarado et al. [44]
Collagen remodeling responds to mechanical stimuli	Indirect (tendon/ligament studies)	Hinz [13]; Humphrey et al. [35]
Fascial memory as a distinct pathological entity	Hypothetical	Proposed in this paper
Stacking fascia represents accumulated fascial memory	Hypothetical	Proposed in this paper; requires histological validation
FHR resets fascial memory via mechanotransduction reversal	Hypothetical	Proposed in this paper; requires controlled trials

## Data Availability

No new data were created or analyzed in this study. Data sharing is not applicable to this article.

## Supplementary Materials

The following supporting information can be downloaded at: https://www.mdpi.com/article/10.3390/ijms27093720/s1.
